# Antibacterial effects of oak bark (*Quercus robur*) and heather herb (*Calluna vulgaris L*.) extracts against the causative bacteria of bovine mastitis

**DOI:** 10.14202/vetworld.2022.2315-2322

**Published:** 2022-09-28

**Authors:** Renāte Šukele, Ingus Skadiņš, Rudīte Koka, Dace Bandere

**Affiliations:** 1 Department of Pharmaceutical Chemistry, Faculty of Pharmacy, Rīga Stradiņš University, LV-1007 Riga, Latvia; 2 Department of Pharmaceuticals, Red Cross Medical College of Rīga Stradiņš University, LV-1007 Riga, Latvia; 3 Department of Biology and Microbiology, Rīga Stradiņš University, LV-1007 Riga, Latvia; 4 Baltic Biomaterials Centre of Excellence, Headquarters at Riga Technical University, Dzirciema Street 16, Riga, LV1007, Latvia

**Keywords:** antibacterial, bovine mastitis, *Escherichia coli*, heather herb, *in vitro*, oak bark, *Staphylococcus aureus*, *Streptococcus*

## Abstract

**Background and Aim:**

Bovine mastitis has a negative impact on animals, and improper antibiotic use has caused an increase in bacterial resistance. Therefore, medicinal plants could serve as an alternative treatment for this condition. Polyphenols have potential as antibiotic agents. Oak bark has long been used as a medicine and has shown antibacterial effects. Moreover, research on heather plant demonstrated that it has antibacterial properties. This study aimed to assess the antibacterial effects of oak (*Quercus robur*) bark and heather (*Calluna vulgaris L*.) herb extracts against common bovine mastitis pathogens.

**Materials and Methods:**

Dried oak bark and heather herb were used to prepare extracts using 30%, 50%, and 70% ethanol and acetone as solvents. Their polyphenol content was determined using the Folin–Ciocalteu method. Bovine mastitis-inducing clinical isolates of *Escherichia coli*, *Streptococcus agalactiae*, *Streptococcus uberis*, *Serratia liquefaciens*, *Staphylococcus aureus*, and reference cultures of *S. aureus* and *E. coli* were used for antibacterial tests. All extracts were screened through a disk diffusion test to ascertain their antibacterial effects, and the minimum inhibitory concentration (MIC) and minimal bactericidal concentration (MBC) were determined for the most effective extracts.

**Results:**

Oak bark extracts had variable antibacterial effects against *S. aureus* and *Streptococcus* strains, but no statistically significant difference was observed in activity against *E. coli*. The disk diffusion test showed that the oak bark extracts obtained using acetone and ethanol at 30% yielded the best results. However, the 70% acetone oak extract alone affected all types of bacteria. Further antibacterial tests of 70% acetone and 30% ethanol oak extracts revealed that the lowest MIC and MBC scores were against *S. aureus* strains and *E. coli* reference cultures. Conversely, the heather herb extracts exhibited broader activity against all types of bacteria, although better results were observed against Gram-positive bacteria. There was also a negative correlation between solvent concentration and antibacterial effect (p < 0.05, r = −0.507). The highest inhibition zone scores and broadest spectrum were observed in samples prepared in 30% ethanol. There was no statistically significant correlation between the phenolic content of plants and their antibacterial effects.

**Conclusion:**

Oak bark and heather extracts could be used as potential antibacterial agents against bovine mastitis pathogens.

## Introduction

Bovine mastitis has been a problem for over 100 years in the dairy industry [1]. Mastitis leads to monetary losses for farmers, which include not only direct milk losses and a decrease in product quality but also treatment, veterinary, and labor costs, making it the most costly disease of dairy cattle. Preventative actions can reduce mastitis cases in dairy farms [2, 3]. Proper teat, animal, and milking hygiene; animal treatment after milking and dry periods; and therapy protocols are considered to be the most effective [1]. Bovine mastitis consists in an inflammation of the mammary gland associated with bacterial infection, which results in decreased milk production and has an overall negative impact on animal welfare [2, 4]. Moreover, infection increases the number of leukocytes and cell death, which, in turn, elevates the somatic cell count in milk. Mastitis can be caused by various Gram-positive and Gram-negative bacteria, both from environmental and contagious sources. The most commonly identified mastitis-causing pathogens in dairy cattle are Staphylococcus aureus, non-aureus staphylococci, Escherichia coli, Corynebacterium bovis, Streptococcus uberis, Streptococcus dysgalactiae, Mycoplasma spp., and Enterococcus spp. [5, 6].

Antibiotic administration is the typical treatment of bovine mastitis. However, this approach yields an inadequate cure rate and leads to the presence of antibiotics in milk [7]. Furthermore, excessive use of antibacterial medicines has led to antibiotic resistance, which triggers new problems in mastitis treatment. Cloxacillin- and oxacillin-resistant staphylococci and streptococci and vancomycin-resistant bacteria have been observed in groups that used antibiotics excessively [8]. Although a Canadian study found that the overall antibacterial agent resistance of mastitis-causing pathogens was low, penicillin- and multiantibiotic-resistant *S. aureus* was observed [9]. A previous study has mentioned persistent intramammary infection caused by virulence alterations in *E. coli* [10]. Reducing the use of antibiotics in husbandry, treating multiresistant bacteria, and implementing preventative measures for antibacterial resistance are key goals of veterinary medicine in Europe [11, 12]. Furthermore, searches for new possibilities for the treatment and prevention of this condition have received much attention recently. Cow vaccination is one of the approaches used to prevent mastitis. Most of the developed vaccines target *S. aureus*, *Streptococcus agalactiae*, and *E. coli* [6]. A review of alternative therapies and preventative treatments for bovine mastitis indicated the possible use of herbals and phytochemicals, bacteriocins, probiotic bacteria, bacteriophages, animals, venoms, nanoparticles, and cytokines [7, 13]. Some herbs and derived bioactive compounds have antibacterial and anti-inflammatory effects, both contributing to the treatment of mastitis. The reported herbal antibacterials belong to plant secondary metabolites: Alkaloids, sulfur-containing compounds, essential oils (terpenoids), coumarins, and polyphenols [14]. Synergy between conventional antibiotics and plant-derived compounds has been observed [15]. In addition, synergy within plant extracts themselves or antibiotics has provided encouraging results, with whole-plant extracts and compound combinations showing greater effectiveness [16, 17]. The diverse mechanisms of the action of polyphenols and their synergic effects with antibiotics were noted as reasons for alternative or complementary therapy for infection treatment [18, 19]. Polyphenolic compounds are a vast chemical group of plant secondary metabolites. More than 8000 of these polyphenolic compounds are found in food and non-food plants. Although polyphenols are diverse and complex in their structure, all of them contain phenyl rings and at least one hydroxyl substituent [20]. There are various classifications for polyphenols, that is, simply dividing them into flavonoid and non-flavonoid groups [20] or diving them into major classes, as follows: Phenolic acids, flavonoids, tannins, stilbenes, and lignins [21]. Phenolic acids can be subclassified into hydroxybenzoic acids (gallic acid and ellagic acid) and hydroxycinnamic acids (caffeic acid, chlorogenic acid, and ferulic acid). The broad group of flavonoids includes flavonols, flavones, isoflavones, fla­vanones, and anthocyanidins [22]. Tannins are usually divided into hydrolysable (gallotannins and ellagitannins) and condensed tannins, also called proanthocyanidins [23]. The lignan structure consists of two phenylpropane units, which are mostly described in medicine as phytoestrogens (ecoisolariciresinol). Stilbenes are made of two phenyl moieties connected by a two-carbon methylene bridge. Resveratrol is the best studied of the stilbenes; however, they are found in low quantities in plants [24, 25].

Oak bark is traditionally used in Europe. The European Pharmacopeia describes it as the cut, dried bark of *Quercus robur*, *Quercus petraea*, and/or *Quercus pubescens*, which contains at least 3% tannins [26]. According to the Committee on Herbal Medicinal Products, oak bark and its preparations are used for the treatment of minor inflammations of the mucosa or skin, hemorrhoids, and mild diarrhea, and are deemed safe. An assessment report also provided an overview of other effects, such as astringent, gastroprotective, antibacterial, antiviral, antiprotozoan, antifungul, antiparasitic, antioxidant, and anticancer activity [27]. The polyphenols identified in *Q. robur* bark include ellagic acid, gallic acid, protocatechuic acid, catechin, and vanillic acid [27]. *Calluna vulgaris* (L.) Hull, or common heather, is a flowering plant that grows in Europe and has aerial parts that contain various polyphenolic substances. This herb is commonly used for medicinal purposes. The polyphenols described in heather herb include aucubin, arbutin, chlorogenic acid, flavonoids, and tannin metabolites [28–30]. Heather herb and oak bark are possible sources of antimicrobial agents. In our study, oak bark was chosen for its well-known high content of polyphenolics – tannins – and its medicinal use, whereas heather herb was selected because of a growing science-based interest in the use of traditional herbs and folk medicine.

This study aimed to assess the possible antibacterial effects of oak bark (*Quercus robur*) and heather herb (*Calluna vulgaris L*.) extracts against common bovine mastitis pathogens.

## Materials and Methods

### Ethical approval

The study is based on the identification of antibacterial properties and chemical composition analysis of medicinal plants without the use of animals therefore, no ethical approval was necessary.

### Study period and location

For the study was conducted from August 2020 to January 2022 at Riga Stradins University’s Department of Pharmaceutical Chemistry and Department of Biology and Microbiology.

### Extract preparation for the determination of total polyphenol content (TPC) and antibacterial screening

The aerial parts of heather herb were collected during flowering in August of 2019 in the Sigulda area, Latvia, it was identified by prof. Dace Bandere, then dried according to the general guidelines. Dried plant material was stored in hermetically sealed dark glass jars until the analysis. Latvia has a moderate climate and seasonal variation. Oak bark (*Q. robur*) was purchased from the AS Švencioniu Vaistažoles vendor, Lithuania, with batch number L1092001. The dried herbals were ground in a mill and sieved through 2 mm sieves. Extracts were prepared using 10 g of plant powder and 100 mL of acetonic or ethanolic solutions at three different concentrations (30%, 50%, and 70%). The plant material was macerated using an orbital shaker (PSU-10i Biosan) at ambient temperature for 80 min. The solvent was removed from the extracts through rotary vacuum evaporation (Heidolph Laborota 4002 control). Each extract was quantitatively moved (dissolved by adding water up to 5 mL) to ambient vials and freeze stored until analysis, that is, TPC and agar disk diffusion tests.

### Extract preparation for antibacterial tests

Plant extracts were chosen corresponding to previous screening data pertaining to susceptibility, that is, the agar disk diffusion test. Dried and ground plant material was extracted using the steps described above. For the determination of the minimum inhibitory concentration (MIC) and minimal bactericidal concentration (MBC), the extracts were freeze-dried by lyophilization (–80°C) after removing the solvent through rotary vacuum evaporation.

### Microbiological cultures

Six bovine mastitis clinical isolates (*E. coli* [ID. V-2019-4], *E. coli* [ID. V-2019-252], *S. agalactiae* [ID. V-2019-171], *S. uberis* [ID. V-2019-243], *Serratia liquefaciens* [ID. V-2019-251], and *S. aureus* [ID. V-2019-256]) were obtained from the Latvia University of Life Sciences and Technology, Research Laboratory of Biotechnology. The antibacterial susceptibility of the clinical isolates of bovine mastitis is described in Table-1. The two reference strains, *S. aureus* (ATCC 25923) and *E. coli* (ATCC 25922), were provided by Riga Stradins University, Biology and Microbiology Faculty.

**Table-1:** Characteristics of clinical isolates of bovine mastitis.

Antibiotic	*E. coli* (ID. V-2019-4)	*E. coli* (ID. V-2019-252)	*S. agalactiae* (ID. V-2019-171)	*S. uberis* (ID. V-2019-243)	*S. liquefaciens* (ID. V-2019-251)	*S. aureus* (ID. V-2019-256)
Amoxicillin	R	R	S	R	R	R
Amoxicillin/clavulanic acid	R	I	S	S	S	S
Ampicillin	R	R	I	S	R	R
Cefotaxime	S	S	S	S	N	N
Cefovecin	S	N	N	N	N	N
Cefotaxime	N	N	N	N	S	S
Ceftiofur	N	S	S	S	S	S
Cephalexin	R	R	I	S	I	I
Enrofloxacin	S	S	R	S	S	S
Erythromycin	N	R	N	N	R	R
Gentamycin	S	N	R	R	N	N
Lincomycin	N	N	R	N	N	N
Neomycin	R	R	N	R	I	I
Novobiocin	N	R	N	R	R	R
Penicillin	R	R	N	N	N	N
Penicillin G	N	N	R	R	R	R
Oxytetracycline	I	I	R	R	S	S
Trimethoprim/sulfamethoxazole	N	N	R	N	N	N

S=Sensitive, I=Intermediate, R=Resistant, N=Not tested, *E. coli=Escherichia coli*, *S. agalactiae*=*Streptococcus agalactiae*, *S. uberis*=*Streptococcus uberis*, *S. liquefaciens*=*Serratia liquefaciens*, *S. aureus=Staphylococcus aureus*

### Determination of total polyphenolic content

The reagents and chemical substances used for these experiments were purchased from Chemo Lab: analytical grade gallic acid, sodium carbonate, and Folin–Ciocalteu reagent. The total polyphenolic content was determent using the Folin–Ciocalteu method with minor modifications [31]. Semi-dry plant extracts were dissolved in 250 mL of distilled water and filtered. Then, 1 mL of this solution was diluted to 50 mL. To 1 mL of the obtained solution, 5 mL of 10% Folin–Ciocalteu reagent and 4 mL of 7.5% sodium carbonate were added. The preparations were kept in the dark and absorbance was measured after 30 min at 765 nm. A gallic acid (c = 0.120 mg/L) solution ranging from 0.0075 to 0.900 mg/mL was used as a standard for the preparation of a calibration curve.

### Antibacterial assays using the agar disk diffusion test

The antibacterial properties of heather herb and oak bark extracts were tested using the standard laboratory method to determine this susceptibility, that is, the agar disk diffusion test.

A standardized bacterial inoculum of each tested bacteria with 0.5 McFarland density was prepared and inoculated using a sterile cotton swab on Mueller-Hinton (MH) (Oxoid, UK) agar or Mueller-Hinton agar with 5% sheep blood (Oxoid) (MHBA). After inoculation, four sterile filter paper disks (5 mm in diameter) were placed on the agar surface and impregnated with 15 μL of heather herb or oak bark semi-solid extracts, which were prepared by evaporating the solvent and were quantitatively moved (dissolved with water up to 5 mL) to vials.

The MH and MHBA agar plates were incubated under suitable conditions in a thermostat (Memmert, Germany) for 18 h at 37°C. Twenty-four hours later, the antibacterial properties of the selected samples were analyzed by measuring the sterile area (diameter) around the disk.

### Determination of the MIC and MBC of the extracts

The MIC of extracts was determined using the broth microdilution method, which is the standard antibacterial susceptibility testing method. Bacterial suspensions of 0.5 McFarland density were used to obtain a final suspension with a bacterial concentration of 106 Colony-forming unit (CFU)/mL.

The quantitative assay of the antibacterial activity was performed in 96-well plates (SarsTEDT, Germany). The dry extract was dissolved in 2 mL of dimethyl sulfoxide. The first extract solution was diluted 10-fold with Mueller-Hinton broth (MHB, Oxoid) and was then used for 2-fold serial dilutions of the compound solutions in a 50 μL volume of Mueller-Hinton broth (MHB, Oxoid). Each well was seeded with 50 μL of the bacterial suspension (10^6^ CFU/mL). The 96-well plates were incubated in a thermostat (Memmert) for 18 h at 37°C. The MIC values were considered as the lowest concentration of the tested compound that completely inhibited bacterial growth in the microdilution wells, as detected by the unaided eye. To determine the MBC, 10 μL of a subcultured sample were inoculated on an MH plate from each microdilution well; one above the MIC value and three below the MIC value. The MH plates were incubated in a thermostat (Memmert) for 18 h at 37°C. The MBC was defined as the lowest concentration that kills all bacteria. Each sample was tested 4 times, and a solution of broth without extract was used as the control.

### Statistical analysis

Data were tabulated using Microsoft^®^ Excel^®^ 2019 MSO (16.0.10382.20010) 32-bit (Microsoft, USA) and analyzed using The IBM^®^ SPSS^®^ Statistics Software (Version 27.0; IBM Cor^p^©, Armonk, NY, USA). Simple descriptive statistics (one-way analysis of variance and the Mann–Whitney–Watt test) were used to investigate the significance of differences between samples. Spearman’s correlation analysis was also used. In all cases, significance was set at p < 0.05.

## Results

The results of the agar diffusion test are provided in Table-1. Regarding the measured diameter of the inhibition zone in mm (±standard deviation), only results over 7 mm were considered as viable. The initial screening of the extracts (Table-2) revealed significant differences (p < 0.05) between the antibacterial effects of oak bark extracts against *S. aureus* and *Streptococcus* strains, whereas no significant difference was detected against *E. coli*. The largest inhibition zones were observed for oak bark extracts prepared using 30% acetone and 30% ethanol. However, the oak extract in 70% acetone alone affected all types of bacteria tested, including *S. liquefaciens*, indicating a broader antibacterial spectrum. The Mann–Whitney–Wilcoxon statistical test was applied to assess differences between the effects of the extracts on bacteria according to the cell wall type. Oak bark extracts prepared using 50% ethanol, 70% ethanol, 50% acetone, and 70% acetone showed statistically significant differences among Gram-positive types of bacteria (*S. aureus*, *S. agalactiae*, and *S. uberis*) compared with Gram-negative bacteria (*E. coli* and *S. liquefaciens*) (p < 0.05). There was no difference in effectiveness between the extracts prepared using 30% ethanol and 30% acetone as a solvent in comparison according to cell wall type (p > 0.05). Overall, the extracts had better antibacterial effects against *S. aureus* strains. There was no significant correlation between the amount of polyphenols in the extracts and their antibacterial effects in either group (p < 0.05). Although there was a positive correlation between ethanol extract type and TPC, a higher ethanol concentration yielded a greater TPC (p < 0.05, r = 0.949), whereas no such correlation was observed in the acetone extract group (p > 0.05). For further analysis, we chose the oak bark extract prepared with 30% ethanol, for its highest scores, and the oak bark extract prepared using 70% acetone, for its broadest spectrum.

**Table-2:** Total phenolic content and inhibition zones by herb and extract solvent type.

Extract by solvent and concentration	Total phenolic content, GAE mg/100 g (+/-SD)	Inhibition zone by extract type, mm (±SD)							

		*S. aureus* V256	*S. aureus* ATCC	*E. coli* V252	*E. coli* V4	*E. coli* ATCC	*S. agalactiae* V171	*S. uberis* V243	*S. liquefaciens* V251
Oak bark									
Ethanol 30	3399 (269)	27.3 (0.9)	26.0 (2.2)	12.0 (1.4)	11.5 (0.6)	10.5 (1.0)	10.3 (0.5)	10.5 (0.6)	0.0 (0.0)
Ethanol 50	3920 (169)	26.8 (2.5)	24.5 (2.1)	12.3 (0.5)	11.3 (1.3)	10.25 (1.7)	10.5 (0.6)	10.5 (0.6)	0.0 (0.0)
Ethanol 70	5462 (410)	25.5 (0.6)	22.0 (3.2)	11.5 (0.6)	10.7 (0.6)	9.8 (2.1)	10.8 (0.5)	10.8 (0.5)	0.0 (0.0)
Acetone 30	2809 (997)	26.5 (1.9)	26.0 (1.4)	12.3 (1.5)	12.5 (0.6)	11.8 (1.3)	10.0 (0.0)	10.0 (0.0)	0.0 (0.0)
Acetone 50	4201 (1061)	25.3 (1.5)	22.3 (1.3)	11.0 (0.8)	10.8 (0.5)	9.7 (0.9)	10.0 (0.0)	10.0 (0.0)	0.0 (0.0)
Acetone 70	4374 (1015)	21.8 (1.0)	22.0 (1.0)	10.5 (1.0)	11.0 (0.8)	11.3 (1.6)	10.0 (0.0)	11.8 (1.0)	10.0 (0.0)
Heather herb									
Ethanol 30	2755 (349)	25.3 (2.2)	18.8 (1.5)	10.5 (1.0)	13.8 (0.9)	10.5 (1.0)	10.5 (1.0)	11.5 (0.6)	11.3 (0.5)
Ethanol 50	3069 (879)	23.5 (1.9)	19.3 (1.7)	10.0 (0.0)	12.5 (0.9)	0.0 (0.0)	10.5 (0.6)	10.0 (0.8)	9.8 (1.3)
Ethanol 70	4368 (351)	20.5 (3.3)	15.5 (1.0)	0.0 (0.0)	9.0 (0.8)	10.0 (1.6)	10.8 (0.5)	0.0 (0.0)	9.3 (0.3)
Acetone 30	3210 (1037)	24.5 (2.1)	18.5 (1.3)	9.5 (0.6)	11.8 (1.7)	10.0 (1.6)	10.0 (0.0)	11.5 (0.6)	11.25 (1.3)
Acetone 50	5106 (756)	23.5 (2.7)	20.0 (0.8)	9.25 (0.9)	11.8 (1.3)	0.0 (0.0)	10.0 (0.0)	11.0 (0.8)	10.8 (0.5)
Acetone 70	5271 (756)	22.8 (4.6)	16.5 (1.3)	0.0 (0.0)	8.5 (0.6)	0.0 (0.0)	11.8 (1.0)	9.0 (1.0)	0.0 (0.0)

*E. coli=Escherichia coli*, *S. agalactiae*=*Streptococcus agalactiae*, *S. uberis*=*Streptococcus uberis*, *S. liquefaciens*=*Serratia liquefaciens*, *S. aureus=Staphylococcus aureus*

The analysis of the extracts of heather herb (Table-2) revealed inhibition zone measurement differences among the different solvent concentrations. The ethanol extracts were the most effective against *S. aureus* strains. The antibacterial effectiveness of the ethanol extracts against the various bacterial strains was as follows (highest to lowest): *S. aureus* V256 > *S. aureus* ATCC > *E. coli* V4 > *S. uberis* V243 > *S. liquefaciens* V251 > *S. agalactiae* V171 > *E. coli* ATCC > *E. coli* V252. Spearman’s correlation statistical analysis showed a negative correlation between solvent concentration and antibacterial effect (p < 0.05, r = –0.507). The highest measurement scores for the inhibition zones (10.5–25.3 mm in diameter) and broadest spectrum were observed in 30% ethanol samples. The acetone-based extracts of heather herb exhibited the following effectiveness: *S. aureus* V256 > *S. aureus* ATCC > *E. coli* V4 > *S. uberis* V243 > *S. liquefaciens* V251 > *S. agalactiae* V171 > *E. coli* ATCC > *E. coli* V252. The best results were observed for the 30% acetone extracts. There was also a correlation between extract solvent concentration and antibacterial effect (p < 0.05, *r* = –0.469) lower concentration of acetone extract had better activity. Analysis of relation between extract solvent concentration and TPC showed correlation (ethanol: p < 0.05, *r* = 0.719; acetone: p < 0.05, *r* = 0.577). Because the TPC was greater in extracts in which the solvent had more organic solvent, whereas a better antibacterial effect was observed at lower solvent concentrations, other extracted active substances might be responsible for the antibacterial effects observed here. Further investigation of the heather herb extracts composition is needed. The MIC and MBC tests were performed using heather herb 30% ethanol extracts.

The heather herb dry extract starting mass was 730.6 mg, that of the oak bark dry extract with ethanol was –493.5 mg, and that of the oak bark dry extract with acetone was –479.2 mg.

The results of MIC and MBC for both extracts are shown in Table-3. The MBC of the oak bark acetone extracts ranged from 3.08 to 24.68 mg/mL, whereas that of the ethanol extracts was 1.49–47.92 mg/mL. For both types of extracts, the lowest MIC and MBC values were noted against *S. aureus* strains and *E. coli* ATCC. When comparing the two types of oak extracts, we found that both yielded similar results, although the ethanol extracts had lower MIC and MBC values against *S. aureus*. The MBC of the heather extracts ranged from 2.28 to 73.06 mg/mL. The lowest MIC and MBC values were observed for *S. aureus* strains, whereas the highest values were observed for *Streptococcus* and *Serratia* bacteria.

**Table-3:** MIC and MBC of oak bark extracts and heather extract.

Bacterial cultures	Oak bark 30% ethanol, mg/mL		Oak bark 70% acetone, mg/mL		Heather herb 30% ethanol, mg/mL	
		
	MIC	MBC	MIC	MBC	MIC	MBC
*E. coli* ATCC	6.16	6.1	5.99	5.99	18.27	18.27
*E. coli* V252	12.34	12.34	11.98	26.96	18.27	18.27
*E. coli* V4	12.34	12.34	11.98	11.98	18.27	18.27
*S. aureus* ATCC	3.08	3.08	1.49	2.99	1.14	4.56
*S. aureus* V256	1.54	3.08	0.78	1.49	1.14	2.28
*S. agalactiae* V171	24.68	24.68	23.96	23.96	18.27	36.53
*S. uberis* V243	24.68	49.4	47.92	35.93	36.50	73.06
*S. liquefaciens* V251	- *	- *	47.92	47.92	73.06	73.06

* Was not tested since showed no effect in disk diffusion test. MIC=Minimum inhibitory concentration, MBC=Minimum bacterial concentration, *E. coli=Escherichia coli*, *S. agalactiae*=*Streptococcus agalactiae*, *S. uberis*=*Streptococcus uberis*, *S. liquefaciens*=*Serratia liquefaciens*, *S. aureus=Staphylococcus aureus*

## Discussion

In this study, six herbal extracts from two plants were tested. The analysis of the results showed that antibacterial effects varied between plant extracts and bacteria types. Oak bark extracts produced larger inhibition zones than did heather extracts. All concentrations of the oak bark extracts had effects on *S. aureus*, *E. coli*, and *Streptococcus* strains, whereas more types of heather herb extracts showed antibacterial activity against *Serratia* spp.

Differences in the chemical composition of the plant extracts and the type of the bacterial cell wall could affect the antibacterial effect. In the case of the oak bark extracts, their effectiveness varied to a great extent according to the bacterial cell wall structure, and generally, they presented better antibacterial effects against Gram-positive bacteria. Similarly, a study of mastitis-inducing bacteria also revealed lower effects against Gram-negative bacteria [32]. *Schinopsis brasiliensis* water extracts had better activity against *S. aureus*, whereas *Caryocar brasiliense* ethanol extracts had the highest activity against *E. coli*, possibly because of a more diverse chemical composition. Because the removal of tannins decreased the antibacterial activity, polyphenols may be responsible for this effect. A study of oak species showed that *Q. robur* had better antibacterial activities than other oak species. Its bark extract contained ellagic acid, gallic acid, protocatechuic acid, and vanillic acid, and antibacterial activities against *Pseudomonas aeruginosa*, *Micrococcus flavus*, and *E. coli*. The antibacterial activities of phenolic standards of the ellagic and caffeic acids were comparable and were higher than those of *Q. robur* and *Quercus macrocarpa* extracts, respectively [33]. An analysis of the chemical composition of an oak bark decoction and the antibacterial properties of the identified substances directly tied the phenolics 1,2,3-trihydroxybenzene (pyrogallol) and 4-propyl-1,3-benzenediol to antibacterial effects. An additional seven active compounds detected in the oak bark decoction exhibited an antibacterial effect to some extent. Thus, the chemical composition of the extract is linked to its antibacterial properties [34]. Considering that, in our experiment, the 70% acetone extract had a broader spectrum, different active substances may have been extracted. A more precise analysis of the constituents of each extract is necessary to determine other variables and the relationship between polyphenols and antibacterial effects.

The heather herb extracts showed a correlation between polyphenolics and antibacterial effects, as well as better antibacterial effects against Gram-positive bacteria, especially *S. aureus*. The antibacterial effects of polyphenols have been described in many studies. Polyphenols, phenolic acids, and their mixtures in plant extracts have been shown to exhibit antibacterial activity against Gram-positive bacteria (*S. aureus* and resistant strains) [18, 35]. Antibacterial tests against foodborne pathogenic and food spoilage bacteria showed that the antibacterial effects of polyphenols are strain-dependent [19]. A literature review described the antibacterial effects of several polyphenolics (flavan-3-ols, flavonols, tannins, and phenolic acids), which exhibited different antibacterial activity against Gram-positive and Gram-negative bacteria, as well as multiresistant strains of bacteria and fungi (*Candida albicans*) [36]. Many studies [28, 37–40]reported that the antibacterial effects of heather herb extracts vary according to bacterial strain and type of solvent used. An analysis of hydroalcoholic heather extracts reported that the MIC values of *S. aureus* and *Staphylococcus epidermidis* were lower than 8 mg/mL, whereas aqueous and alcoholic extracts showed higher values. The alcoholic heather extract yielded the highest MIC values in *P. aeruginosa*, *E. coli*, *Klebsiella pneumoniae*, and *C. albicans* [37]. The MIC values of heather methanolic extracts in *S. aureus* and *Staphylococcus hominis* were 0.1 μg/mL. The hydroalcoholic extract contained quercetin, kaempferol, and myricetin derivatives [38]. A screening of dry extracts (solvent, ethanol 70%) of heather herb showed weak antibacterial activity against *S. aureus*, but no activity against *E. coli* strains [39]. The chemical analysis of the heather ethanol extract identified phenolic compounds, flavonoids, alkaloids, saponins, and antimicrobial activity against *Agrobacterium tumefaciens*, *Erwinia* spp., *K. pneumoniae*, and *P. aeruginosa*, but did not show activity against *E. coli* and *Proteus* spp. [40]. The study of three types of heather extracts and 30 pathogenic bacteria strains showed the strongest antibacterial activity against *Proteus vulgaris*, whereas the activities on *E. coli* and *Enterococcus faecalis* strains were similar and lower. Compared with ethyl acetate and water extracts, the aqueous extract displayed the strongest effect against all tested bacterial strains, although the ethyl acetate extract had better activity against *P. vulgaris* [28]. The observed data and the results of the previous studies reviewed here suggest that the solvent used for extraction and its concentration affects the antibacterial effect because of changes in the chemical composition of the extracts, as discussed by Vučić *et al*. [28] and Bubonja-Šonje *et al*. [41]. Because there is no standardized method of polyphenol extraction for all plants and herbs [42], various compositions and concentrations of extraction solvents, as well as the effects of temperature and extraction time, need to be considered when comparing study results.

The standardization of MIC levels for medicinal plants has been discussed by many authors [41–44]. There is no unanimity regarding the levels of inhibition for plant extracts. Some researchers suggest using the same criteria as those applied for antibiotics (MIC, 0.01 and 10 μg/mL). Conversely, many authors consider these values to be too low and propose accepting higher levels of inhibition than those adopted for commercial antibiotics. Overall, herbal products are considered antibacterial when the MIC is 100 μg/mL for isolated compounds and 1000 μg/mL for plant extracts [41]. Depending on the author, the MIC classification of crude plants or their extracts varies from <400 to 500 μg/mL for strong antimicrobial effect, 800–1500 mg/mL for moderate effect, and >800 to 1600 μg/mL for a weak effect. However, these limits are considered too high by some researchers [41, 43]. An effectiveness limit of 10 μg/mL for isolated compounds and 100 μg/mL for extracts was proposed by Ríos and Recio [44]. Considering that there is no agreement on MIC lowest or highest level limits for herbal products, the oak bark and heather extracts had either a strong to moderate antibacterial effect, or no considerable effect if the limit is set according to Ríos and Recio [44].

## Conclusion

Oak bark and heather herb are natural sources of polyphenolic compounds. The data generated in this study showed that the type of solvent affected the extracted amounts of polyphenols and the antibacterial effects of the extracts. The results of this study showed that oak bark and heather herb could be potentially used as antibacterials in the case of bovine mastitis, as well as for multi-antibiotic-resistant strains. For oak bark, the highest bacterial inhibition zone scores were observed for 30% ethanol extracts, whereas the broadest spectrum was detected for 70% acetone extracts. For both types of extracts, the lowest MIC and MBC values were noted against both *S. aureus* mastitis isolated strain and *E. coli* reference strain. For heather herb, the best antibacterial effects were observed using the 30% ethanol extract against *S. aureus* strains, whereas the weakest effects were observed against *Streptococcus* and *Serratia* bacteria. In the case of oak bark ethanol extracts, there was a correlation between the yield of polyphenols and their antibacterial effects, whereas no such correlation was observed for the acetone extracts of oak bark or heather herb extracts. Thus, further investigation of the active substances of oak bark and heather herb extracts and their connection to antibacterial effects is necessary.

## Authors’ Contributions

DB and RK: Conceptualized the aim of the study and design. RS and IS: Designed and performed the experiments. RS: Analyzed the data, wrote and edited the manuscript. DB, IS, and RK: Supervised the study and corrected the manuscript. All authors have read and approved the final manuscript.
